# Characterization of HIV-1 Transmission Clusters Inferred from the Brazilian Nationwide Genotyping Service Database

**DOI:** 10.3390/v14122768

**Published:** 2022-12-12

**Authors:** Paula Andrade, Ighor Arantes, Amilcar Tanuri, Gonzalo Bello, Tiago Gräf

**Affiliations:** 1 Laboratório de AIDS e Imunologia Molecular, Instituto Oswaldo Cruz, Fundação Oswaldo Cruz, Rio de Janeiro 21040-360, Brazil; 2 Laboratório de Virologia Molecular, Departamento de Genética, Instituto de Biologia, Universidade Federal do Rio de Janeiro, Rio de Janeiro 21941-902, Brazil; 3 Laboratório de Virologia Molecular, Instituto Carlos Chagas, Fundação Oswaldo Cruz, Curitiba 81350-010, Brazil

**Keywords:** HIV-1, transmission dynamics, phylogenetic clusters, Brazil

## Abstract

The study of HIV-1 transmission networks inferred from viral genetic data can be used to clarify important factors about the dynamics of HIV-1 transmission, such as network growth rate and demographic composition. In Brazil, HIV transmission has been stable since the early 2000s and the study of transmission clusters can provide valuable data to understand the drivers of virus spread. In this work, we analyzed a nation-wide database of approximately 53,000 HIV-1 nucleotide pol sequences sampled from genotyped patients from 2008–2017. Phylogenetic trees were reconstructed for the HIV-1 subtypes B, C and F1 in Brazil and transmission clusters were inferred by applying genetic distances thresholds of 1.5%, 3.0% and 4.5%, as well as high (>0.9) cluster statistical support. An odds ratio test revealed that young men (15–24 years) and individuals with more years of education presented higher odds to cluster. The assortativity coefficient revealed that individuals with similar demographic features tended to cluster together, with emphasis on features, such as place of residence and age. We also observed that assortativity weakens as the genetic distance threshold increases. Our results indicate that the phylogenetic clusters identified here are likely representative of the contact networks that shape HIV transmission, and this is a valuable tool even in sites with low sampling density, such as Brazil.

## 1. Introduction

The human immunodeficiency virus type 1 (HIV-1), particularly the M group, has been diversifying into different subtypes (A to D, F to H, J and K) as it has spread through the human population over the years, causing one of the most significant emerging infectious diseases of the 20th century–AIDS—in terms of affecting the human species [1]. In Brazil, the universal access to antiretroviral treatment (ART) since middle of the 1990s has been successful in containing the AIDS epidemic, however, HIV transmission has stabilized in high values [2]. From 2017 to 2019, an average of 45,000 new cases per year were recorded, with an average of 12,389 new cases in women and 32,409 in men (male: female = 2.6). In this period, men having sex with men (MSM) contribute to approximately 47% of the new HIV infections in men and 33% of all reported HIV cases in Brazil [2]. Currently, it is estimated that approximately 1 million individuals live with HIV in Brazil [3].

Molecular epidemiological studies in Brazil show that three subtypes are responsible for almost 90% of the HIV infections [4]. HIV-1 subtype B is prevalent in the country, except in the southern region [5], with this being the most frequent viral form in 25 of the 27 Brazilian states, reaching the highest level of prevalence in the northern states of Amazonas (91.2%) and Roraima (97.4%) [4]. A different scenario is observed in the southern region where subtype C is found to be the dominant subtype in Santa Catarina (66.2%), Rio Grande do Sul (44.7%) and Paraná (36.8%) [4]. Unlike HIV-1 subtype C, which is highly concentrated in the southern region, subtype F1 is more evenly dispersed across Brazilian states and is responsible for ~9% of infections in the Brazilian territory [5]. The states with the highest prevalence of subtype F1 are Pernambuco, Minas Gerais and Espírito Santo [4].

The high mutation rate of HIV-1 due to the absence of viral reverse transcriptase proofreading [6], combined with the fact that the virus causes a chronic infection, creates a unique viral population in each individual that changes during the course of the infection. This viral population descends from some genetically very similar founder viruses, which were selected shortly after the transmission event and harbor genetic signatures from the viral population of the “donor” individual, allowing the reconstruction of viral transmission networks from the genetic data [7]. In summary, when groups of HIV sequences isolated from different patients have a high degree of genetic similarity, they are connected by a common source, a direct or a short chain of transmissions [8]. Thus, the genetic distance (GD) between HIV sequences is the most commonly used parameter to identify transmission clusters. The GD cutoff, however, is a user-defined parameter that usually varies between 1% and 5% depending on the nature of the sampling and the aims of the study [9].

Phylogenetic techniques have been widely applied to identify HIV transmission networks within populations in different countries and epidemic scenarios [10,11,12,13]. These studies, when associated with epidemiological and clinical information from the sampled individuals, can reveal important details about the dynamics of HIV-1 transmission, such as the role of different risk groups in the spreading of the virus [14,15], the impact of early diagnosis and ART on decreasing transmission [16] and near real-time monitoring of HIV outbreaks to inform the public health response [17]. To increase the power of the identification of transmission networks in a phylogenetic tree, high sample coverage combined with clinical and patient demographic data is crucial [18]. In developed countries, HIV genetic sequences are routinely generated from recently diagnosed individuals to monitor the transmission of drug resistance mutations (DRM) prior to ART onset. In low- and medium-income countries, by contrast, such type of services are rare and studies on HIV transmission clusters rely mostly on cohorts or convenient sampling [19,20,21]. Until the current work, the study of HIV transmission networks in Brazil was limited by small and localized sampling [22] or publicly available sequences that were sparsely collected and poorly annotated with relevant demographic features [21].

In Brazil, the public health system (SUS) offers the genotyping service to individuals failing ART, HIV positive pregnant women and children born with HIV. This service is deployed by the National Genotyping Network (RENAGENO), which has been organizing and curating the largest HIV nucleotide sequence database in Brazil since the early 2000s, representing an important data source for investigating the virus transmission dynamics in the country. In this work, we analyzed the RENAGENO databank using phylogenetics to identify transmission clusters and performed several network and statistical analyses to characterize the clusters. Since the RENAGENO dataset is mostly composed by individuals in the chronic state of the infection, which makes the reconstruction of the past epidemiological links difficult, our main goal was to assess the potential value of this source of data when inferring the contact network and, ultimately, to identify factors capable of improving policies for monitoring and controlling the HIV/AIDS epidemic in Brazil.

## 2. Materials and Methods

### 2.1. The RENAGENO Dataset, Quality Control and Ethical Approval

The department of Diseases of Chronic Conditions and Sexually Transmitted Infections, from the Department of Health Surveillance, Ministry of Health, provided us access to the RENAGENO database that has approximately 53,400 nucleotide sequences of protease (PR) and reverse transcriptase (RT) genes generated from samples collected between 2008 and 2017. These genes are the main point of action of antiretrovirals and they are also the place where resistance mutations occur, thus being the focus of the genotyping service. In addition to the HIV-1 nucleotide sequences from sampled patients from all over Brazil, epidemiological data on patients, such as gender, date of birth, year of education, color/race, state and city of residence, were also provided. Nucleotide sequences were analyzed using the Quality Control tool from the Los Alamos HIV Database (available at: https://www.hiv.lanl.gov/content/sequence/QC/index.html, accessed on 1 March 2019) that evaluates quality parameters, such as the presence of hypermutated sequences and identical sequences, and sequences that did not pass this quality control were excluded from the database. In addition we also excluded sequences with multiple indels, sequences of patients aged 14 and under (to avoid biases related to vertical transmission when inferring transmission clusters) and patients’ sequences where there was no date of birth included. When multiple sequences from the same patient were available, only the first one was analyzed. This work was approved by the Research Ethics Committee of the Instituto Gonçalo Moniz/Fiocruz-BA under registration number 15300719.5.0000.0040. An informed consent waiver was obtained from the ethics committee.

### 2.2. HIV-1 Group M Subtyping

Approved sequences were classified into HIV-1 M subtypes through the online tools COMET [23], REGA Subtyping Tool v3.0 program [24] (as available at: https://www.genomedetective.com accessed on 1 March 2019) and RIP (available at: https://www.hiv.lanl.gov accessed on 1 March 2019). Subtype assignment was defined when two or more tools agreed with each other. When there was disagreement between the subtype assigned by these tools, the classification was made by the construction of phylogenetic trees, inferred through the maximum likelihood method in the IQ-TREE program v.2.1.2 [25] using the platform CIPRES Science Gateway [26]. More details about RENAGENO dataset HIV-1 subtyping are available in Gräf et al., 2021 [4].

### 2.3. Phylogenetic Analysis

We analyzed the Brazilian HIV-1 epidemic’s most relevant subtypes B, C and F1, as previously reported [4]. For subtype B, due to the large number of sequences (*n* = 30,434), we firstly performed a classification among the four main clades of subtype B national circulation. This was completed by the evolutionary placement method [27], available in RAxML [28], which can rapidly estimate the position of new query sequences in a reference phylogenetic tree without reconstructing the whole tree. For our purpose, we used a subtype B reference tree and dataset, as described in [29], reducing the RENAGENO subtype B dataset by approximately 50%. The sequences of the three subtypes were aligned separately using MUSCLE algorithm [30] and visually inspected in AliView program [31]. Key codons associated with ARV resistance [32] were masked from the alignments to avoid clustering of individuals due to convergent evolution under selective pressure generated by similar ART history. Transmission networks were reconstructed separately for each of the three subtypes analyzed. To do so, maximum likelihood trees (ML) were constructed using the IQ-TREE program and the best substitution model was estimated with Model Finder [33], as implemented in IQ-TREE. The branch support was calculated initially with the ultrafast bootstrap method with 1000 replicates. Subsequently, these replicates were used to calculate branch support with the transfer bootstrap expectation (TBE) method [34] using the command line tool, “Booster” (open source, available at https://github.com/evolbioinfo/booster accessed on 1 March 2019).

### 2.4. Clusters Identification

The resulting ML trees and the sequence alignments were submitted to the Cluster Picker program [35], which identifies and selects clades in the phylogenetic tree based on branch support and maximum pair-wise genetic distance of the grouped sequences. We applied a minimum of 0.9 TBE support and intra-clade maximum genetic distance (hereafter referred to as GD) values of 1.5%, 3.0% and 4.5%. Number and size of the identified clusters and clustering rates were then comparatively described among the three different analyzed subtypes and GDs. We chose to test different GD cutoffs to investigate how much the networks change in composition and size by altering this parameter. Since our dataset was composed mostly by patients in virologic failure, they are likely to be long-term infected with highly divergent virus populations. Thus, using a range of GD cutoffs allowed us to better describe the behavior of the networks in this very specific sampled population.

### 2.5. Statistical Analysis

To identify predictors of phylogenetic clustering, the odds ratio (OR) of patient demographic features, such as sex, years of education, race and age, was calculated by fitting a binary logistic regression model. We also assessed the correlation between sampling period (2008–2013 and 2014–2018) and clustering. These two periods were chosen due to changes in the protocol of treatment for HIV/AIDS, recommended by the Brazilian Ministry of Health, which, from 2014 onwards, determined that the treatment should be started immediately after the diagnosis and not only for individuals with advanced chronic infection (CD4^+^ T cells <200–500 cells/uL). As a result, it is expected that an increasing number of patients will fail with therapy and need the genotyping service, increasing the number of sequences in the RENAGENO database and potentially impacting clustering rates. Statistical calculation of OR were performed using the packaged Arm in R program (available at: https://www.R-project.org/ accessed on 1 March 2019) and the baseline for the OR comparisons was defined as the more frequent observed feature.

To quantify the tendency of individuals to be connected to each other based on the similarity of demographic features, we calculated the assortativity coefficient in the clusters generated for the three subtypes and GDs. The value of assortativity varies between 1 (completely assortative, i.e., clusters are 100% defined by the analyzed feature) and −1 (completely disassortative, i.e., similar individuals do not cluster together), where an assortativity coefficient of 0 means that the feature has no impact in clustering. Assortativity was calculated using the Igraph package v.1.3.5 [36] available in R. To measure whether the assortativity values of the phylogenetic clusters deviated from an expected null distribution, we calculated the assortativity coefficient from the randomization (1000×) of the demographic features associated with each individual belonging to the identified clusters.

We further explored how geographic distance between patients’ city of residence impacted in cluster formation. To do so, within-cluster geographical distance was calculated by applying the Vincenty ellipsoid method, which calculates the straight-line distance between two points corrected by the earth curvature (available at the geosphere R package). The geographical coordinates of the patient’s city of residence was used and results were compared among GDs and HIV-1 subtypes analyzed.

## 3. Results

### 3.1. Clusters’ Frequency and Size Description

After data cleaning process, we analyzed 5328 subtype C sequences, 4423 subtype F1 sequences and 14,628 subtype B sequences from all the Brazilian regions (Table 1). Considering the three subtypes together, the percentage of sequences in clusters ranged from 1.5% to 2.4% at GD of 1.5%, from 7.7% to 9.3% at GD of 3.0%, and from 19.8% to 23.7% at GD of 4.5%. Regarding clusters sizes, most of the identified clusters were pairs of individuals, accounting for an average of 98.7%, 88.6% and 76.4% of all subtypes clusters at GDs of 1.5%, 3.0% and 4.5%, respectively (Table 1 and Supplementary Figure S1).

### 3.2. Clustering Odds

**Figure 1:**
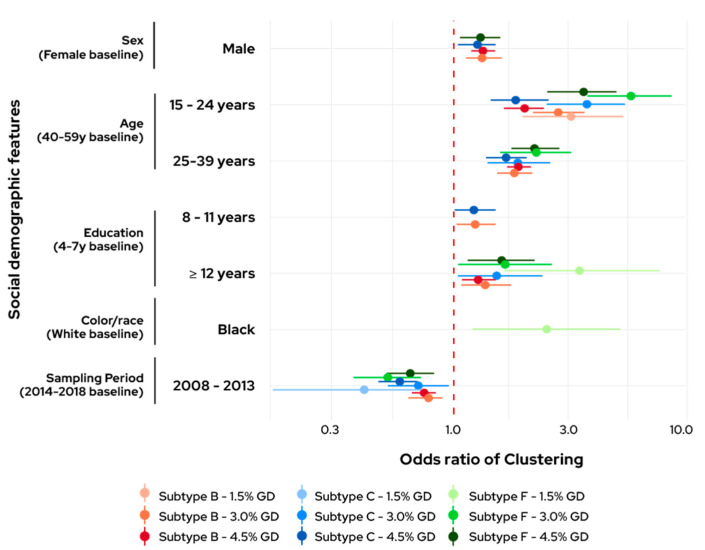
Odds ratio and 95% confidence interval comparing clustered and non-clustered individuals. Only statistically significant (*p* < 0.05) associations are shown.

High ORs in a phylogenetic cluster were observed for young individuals, aged 15 to 24 years, for the three subtypes, and this association is stronger at smaller GDs, tending to decrease as the GD increases, but it still remains statistically relevant, compared to the baseline, 40–59 years (Figure 1). Individuals aged 25–39 years also showed statistically significant association with clustering, although with smaller ORs than younger individuals. The clusters of HIV-1 subtype F1 showed the strongest association between age and clustering for all GDs.

Individuals with more years of education presented higher OR in a cluster, when compared to baseline subjects (4–7 years of education). We also observed that men tend to cluster more than women with very similar OR values among the subtypes (mean OR of 1.7), but we could only detect it at 4.5% GD for all subtypes. When analyzing the sampling period, we observed a negative association (OR, 0.51–0.72) between being sampled in the 2008–2013 period and being clustered. Regarding race, we only observed a significant association for subtype F1 at 1.5% GD, where black individuals showed higher OR, when compared to the baseline white individuals.

### 3.3. Assortativity Analysis of the Networks

**Figure 2:**
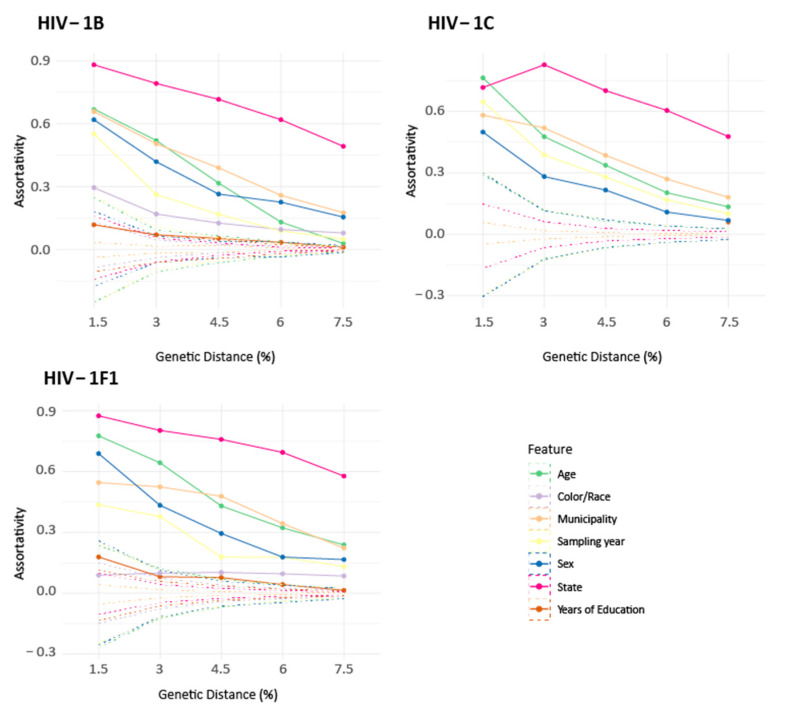
Assortativity coefficient for patients’ demographic features in same phylogenetic clusters. Assortativity (solid lines) and the null distribution (dashed lines) were calculated in clusters identified for HIV-1B, HIV-1C and HIV-1F1 in five different genetic distances.

Assortativity coefficient calculation revealed that most of the demographic features analyzed positively impact on cluster formation. In other words, individuals with similar demographic features tend to cluster together (Figure 2). However, we also observed a gradual decrease in the assortativity coefficient as the genetic distance increases, approaching the null distribution. To better explore the effects of the GD parameter on the assortativity of clusters, we selected and analyzed clusters with GD 6.0% and 7.5%, revealing that even with the maximum GD tested, most of the assortativity values remained outside the null distribution.

Among the individuals´ features submitted to assortativity analyses (i.e., age, race, municipality of residence, state of residence, sampling year, sex and years of education), those with the greatest assortative potential are related to geographic distance, such as the state and municipality of residence. State of residence showed the highest assortativity coefficients for all subtypes in all tested GDs, ranging from values close to 0.9 in 1.5% GD to ~0.5 in 7.0% GD (Figure 2). An exception was observed for subtype C in 1.5% GD, which may be related to the very small number of clusters identified (N = 40) and a spurious assortativity coefficient calculation. As expected, the municipality of residence is less assortative than the state of residence, although it is still a significant feature for clustering even in 7.0% GD. Another highly assortative feature is age, revealing that HIV-1 transmission events occur more often between individuals with similar age, which might be shaped by how sexual relationships are generally established.

Race and years of education are very interesting characteristics to analyze regarding cluster assortativity, since they may be related to the individual´s social class. However, the RENAGENO dataset has a low degree of data completeness for these characteristics (30–44%); thus, we could only consistently calculate assortativity coefficients for subtype B and F1. We observe that both race and years of education might be important demographic characteristics impacting cluster formation and HIV-1 transmission, with some differences among subtype B and F1. For subtype B, race was estimated to be more assortative than years of education, the latter showing only slightly higher values than the null distribution (Figure 2 and Supplementary Table S1), while for subtype F1, years of education seem to play a more important role in cluster formation than race. The latter presents table values across the different GDs, which might be caused by spurious calculations in low GDs, where the number of clustered individuals is small for subtype F1.

### 3.4. Spatial Distribution of the Clusters

**Figure 3:**
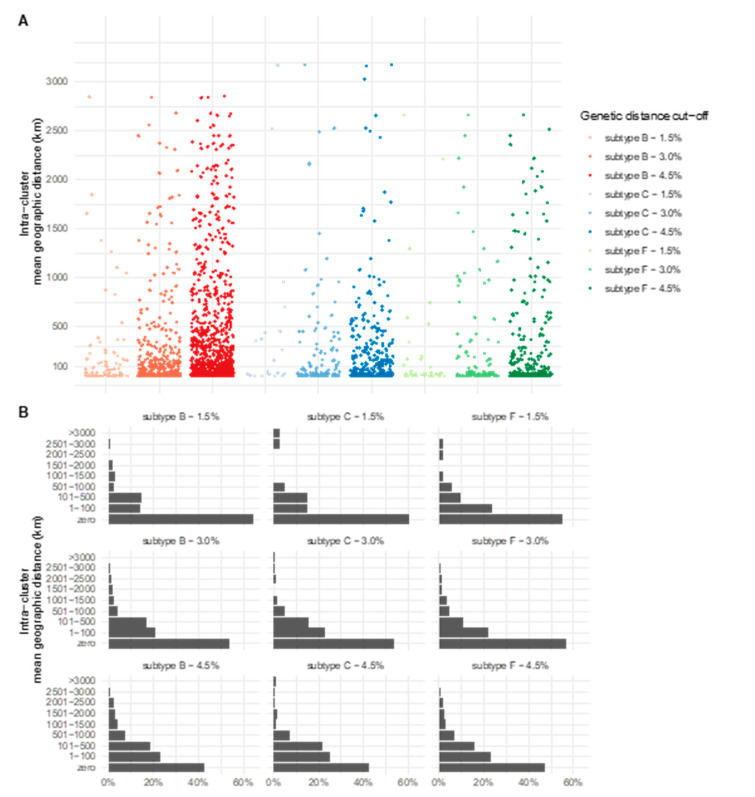
Intra-cluster mean geographic distance for subtypes B, C and F1 in different genetic distance cut-offs. (**A**) Scatter plots showing all clusters identified in each analysis and vertically positioned according to the mean intra-cluster geographic distance (Y-axis). (**B**) Histogram showing the frequency of clusters for categorical groups of mean intra-cluster geographic distances. Zero means that all individuals live in the same municipality.

Once we observed high assortativity for the individuals´ place of residence, we decided to further explore the geographic distance between them. We calculated the average distance among the cities of residence of each individual in a cluster (Figure 3). For all subtypes and GDs, the majority of the clusters were composed by individuals living in the same municipality (0 km of intra-cluster mean geographic distance). As the GD increases, clusters become more geographically dispersed; however, same-municipality clusters still represented more than 40% of all the clusters in all the GDs and subtypes analyzed (Figure 3B). The largest distances (>3000 km) were observed in three clusters of subtype C, representing individuals living in cities located in states at opposite ends of Brazil, such as Maranhão and Rio Grande do Sul, Pará and Santa Catarina, and Pará and Rio Grande do Sul. No significant difference was observed among HIV-1 subtypes regarding clusters’ spatial spread.

### 3.5. Male Clusters Analysis

**Figure 4:**
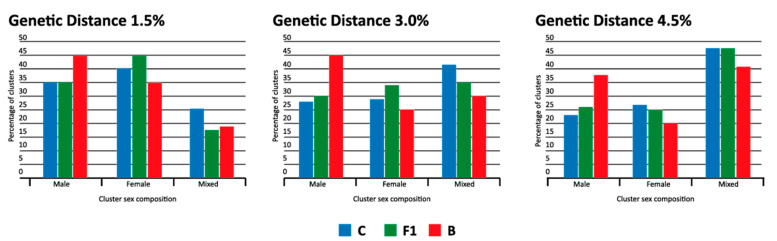
Percentage of clusters composed only by men or women and mixed clusters (clusters composed by men and women), for subtypes B, C and F1, identified with three genetic distances.

Finally, we performed a detailed analysis of clusters composed by individuals of the same sex. This feature was also highly assortative (Figure 3), and we observed that this is due to the high frequency of clusters composed only by men or women. In 1.5% GD, mixed clusters (clusters composed by men and women) comprised only 18–25% of the clusters in the three subtypes, but this proportion increases with genetic distance, reaching 41–49% of clusters in 4.5% GD (Figure 4). Surprisingly, in subtypes C and F1, clusters composed only by women were more frequent than clusters composed only by men, while for subtype B, exclusive male clusters were more frequent than female ones.

**Figure 5:**
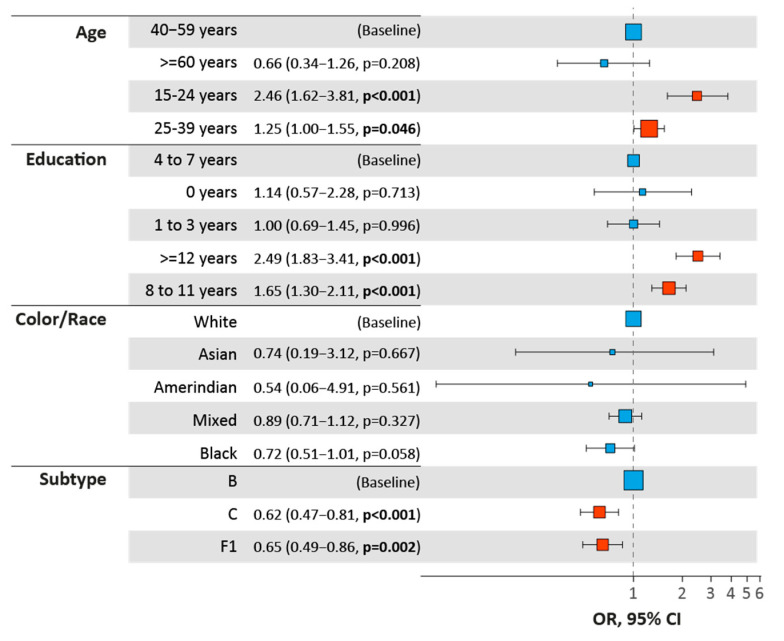
Odds ratio of males clustering with men, compared to males clustering with women in clusters identified with 4.5% GD.

To better characterize the population in male clusters, we performed OR analysis, comparing men in male clusters with men clustered with women, as identified with the 4.5% GD cut-off, thus providing more clusters to be analyzed. Significant OR was observed for young men, more years of education and subtype (Figure 5). As expected by the analysis of frequency of male clusters (Figure 4), subtypes C and F1 were negatively correlated to the presence of male clustering with men, in comparison with subtype B.

## 4. Discussion

This study analyzed HIV-1 sequences generated by RENAGENO, the Brazilian national HIV-1 genotyping service. From a database composed of almost 50,000 HIV-1 pol sequences and their demographic data, we analyzed the three main subtypes (B, C and F1) responsible for ~90% of HIV-1 infections in Brazil [4]. To assess the usefulness of such a dataset to study HIV-1 transmission clusters, three different genetic distances (GD) were applied to identify clusters in phylogenetic trees. Then, we calculated the odds ratio (OR) and the assortativity coefficient to understand the demographic composition of such clusters and how relaxing the GD parameter in the cluster identification process impacted it.

Our study has two main weaknesses, both related to the nature of the RENAGENO dataset. Firstly, few patients’ demographic and clinical data were available and for some features, such as years of education and race, data completeness was low. This prevented us from testing important hypotheses, for example, if higher viral load and date of diagnosis were associated with being part of a transmission cluster or to better characterize MSM clusters. Secondly, the RENAGENO dataset was mainly composed of chronically infected individuals already undergoing treatment failure. Therefore, a large amount of genetic divergence might have accumulated in the patient’s viral population, hampering the identification of large clusters with epidemiologically linked individuals whose transmission event might have occurred years in the past. Although we have analyzed the largest Brazilian HIV-1 sequence database described up to date, a high proportion (>75%) of clusters identified were dyads even in 4.5% of GD (Supplementary Figure S1). Clusters with >5 sequences made up <5% of all clusters, and the largest cluster identified had only nine sequences.

Despite these sampling limitations, we observed a negative association (OR, 0.51–0.72) between being sampled in the 2008–2013 period and being clustered. This might be due to the change in the Brazilian therapy initiation protocol in 2014 that started to include all HIV-positive individuals, irrespective of the CD4^+^ T cell counts. This resulted in the reduction in time between infection with the HIV-1 virus and the sequencing of the virus, since before 2014, only individuals with clinical definition of AIDS were directed to treatment with ART, that is, individuals with years of infection. However, after 2014, all HIV-positive individuals were directed to ART, thus including patients with a short time of viral infection. The GD criteria used here to identify transmission clusters has the tendency to detect recent transmission events [37], and different methods that are not based on a genetic threshold [38,39] should be tested in future studies analyzing datasets of chronic-infected patients.

According to the 2021 HIV/AIDS epidemiological bulletin, provided by the Brazilian Ministry of Health [2], men between 15–24 years old are the unique age group in which AIDS incidence increased between 2010 and 2020. Complying with this data, our results show high OR values of clustering for individuals of 15–24 years for the three subtypes. This association is stronger in smaller GDs and decreases as this parameter is relaxed. Considering that, at a short GD, clusters are mainly composed by individuals recently infected, our results suggest that the 15–24 age group is at high risk of being infected by HIV. As GD increases (relax), older individuals, who are likely to have been infected for a longer time, tend to be added to clusters, decreasing the OR values of the 15–24 age group. This is also supported by the assortativity coefficient of age that decreases as GD increases. When observing individuals aged 25–39 years, OR values are smaller compared to the OR values of the group aged 15–24 years, also suggesting that transmission events occur more frequently among younger individuals. Complementing the basic profile of the individuals with increased odds to cluster, men showed higher OR than women, and among these men our focused analysis in the male clusters revealed that young men presented higher odds of being in a male cluster. Since we did not have access to patients’ sexual behavior data, we used the male clustering profile as a surrogate to infer MSM transmission chains. These results also agree with the Brazilian Ministry of Health epidemiological data, highlighting that this population should be prioritized by health policies. The analysis of the male clusters also revealed that subtype B is positively associated with the MSM transmission chains, a relationship that might be explained by the founder effects of introducing different viral subtypes in partially segregated transmission chains. In Brazil, this scenario was observed by several other studies comparing subtype B and C epidemics, as reviewed in [40].

Significant OR values for clustering related to individuals with more years of schooling (8–11 years and >12 years) might be an indication that these groups of individuals are more likely to seek medical attention at the signs of virological failure and disease recurrence, and to comply with ART follow-up exams. In this way, these individuals could be genotyped as soon as the therapeutic failure occurs, thus increasing their representation in the RENAGENO sampling. Therefore, it may not mean that individuals with high schooling are a group with high HIV transmission, but instead a group of high sequencing rates. Previous studies have shown that genetic clustering methods can be biased to detect clusters of individuals sampled and sequenced soon after the infection and that time since infection would be the main covariate to explain odds of clustering [37,41]. Unfortunately, the date of first diagnosis was not available among the patients’ metadata in the RENAGENO database, and we could not control our analysis for this variable.

Our analyses on the assortativity coefficients of clusters were inversely proportional to the GDs; therefore, the smaller the GD of the clusters, the greater the value of the assortativity. This suggests that at smaller GDs, the phylogenetic clusters approach to the real contact network was formed by, for example, individuals of the same city, in the same age group and of similar social class. A previous study comparing the composition of clusters inferred by genetic methods and the naming partner approach showed a higher degree of agreement in GDs of 1–2% [42]. The most assortative features analyzed here were the ones related to geographic distance. It is expected that individuals from the same municipality or from nearby locations and even within the same state, have higher odds of being part of the same contact network due to the ease of an encounter. As the physical distance increases, the probability of encounter between individuals decreases, making it sporadic and difficult to be sampled among the sequenced individuals. Our results on the average intra-cluster geographic distance complement these findings, showing that even in 4.5% GD, more than 40% of clusters are composed exclusively by individuals living in the same city.

The assortative coefficient concerning features, such as age, race and years of education, can be informative to understand how HIV transmission networks are shaped by social class and demography of the population. Our results show that they do impact in cluster formation and this can be detected by phylogenetic methods of inferring HIV transmission networks. Age was an especially assortative feature, with values comparable to those of the municipality and state of residence. Our results suggest that the main bulk of HIV transmissions occur among individuals with similar age and that age disparity in sexual relationships is not an important contributor to HIV transmission in Brazil, opposing the scenario found in some African countries with high burdens of AIDS [19,43]. Although patient data for years of education and race had low degrees of completeness, our analyses could detect that these features are also important in cluster formation, with assortative values above the null distribution for almost all analyzed GDs and HIV subtypes. The assortative coefficient calculated for race in subtype B clusters with 1.5% GD was similar to the mean value found in several prior network studies analyzing HIV patient interview data (0.3 vs. 0.45, respectively) reviewed by Rothenberg et al. 2007 [44]. These results confirmed previous literature about the assortative selection of partners by race [45,46] and shows that phylogenetic-based methods are useful in detecting such network patterns. Our results on the assortativity of years of education show a smaller role of this feature in cluster formation but also agree with other studies [40].

## 5. Conclusions

Our study contributes to a better understanding of the features shaping network formation and highlights that young (15–24 years old) men are the most vulnerable group to HIV transmission in the Brazilian epidemic scenario. Our results broadly agree with epidemiological data and network studies analyzing HIV patient interview data, showing the value of the phylogenetic approach to studying viral transmission networks in both middle- and low-income countries, where sampling is far from optimal. However, due to the chronic infection state of the majority of the patients sampled in this dataset, the identification of large transmission clusters was restrained, limiting the applicability of phylodynamics when inferring important epidemiological parameters, such as clusters’ reproductive number and growth rate.

## Figures and Tables

**Table 1:** Description of clusters identified for the HIV-1 subtypes.

	Subtype C (*n* = 5328)			Subtype F1 (*n* = 4423)			Subtype B (*n* = 14628)		
	**1.5% GD**	**3% GD**	**4.5% GD**	**1.5% GD**	**3% GD**	**4.5% GD**	**1.5% GD**	**3% GD**	**4.5% GD**
N of cluster	40	194	453	51	182	379	138	637	1494
N of clustered individuals	80	409	1054	105	401	933	276	1362	3467
Clustering rate	1.5%	7.7%	19.8%	2.4%	9.1%	21.1%	1.9%	9.3%	23.7%
Cluster size (% per cluster)									
2 individuals	40 (100%)	178 (91.8%)	355 (78.4%)	49 (96.1%)	155 (85.2%)	274 (72.3%)	276 (100%)	566 (88.9%)	1174 (78.6%)
3 individuals		13 (6.7%)	71 (15.7%)	1 (2.0%)	19 (10.4%)	69 (18.2%)		60 (9.4%)	224 (15.0%)
4 individuals		2 (1.0%)	13 (2.9%)	1 (2.0%)	7 (3.9%)	19 (5.0%)		7 (1.1%)	60 (4.0%)
5 individuals			8 (1.8%)			10 (2.6%)		2 (0.3%)	19 (1.3%)
6 individuals		1 (0.5%)	3 (0.7%)		1 (0.6%)	2 (0.5%)		2 (0.3%)	10 (0.7%)
7 individuals			3 (0.7%)			2 (0.5%)			4 (0.3%)
8 individuals						1 (0.3%)			3 (0.2%)
9 individuals						2 (0.5%)			

As the GD increased, larger clusters were identified, despite still being rare. Clusters formed by at least five individuals represented only an average of 0%, 0.6% and 3.3% in GDs of 1.5%, 3.0% and 4.5%, respectively. The largest cluster found was composed by nine individuals infected with subtype F1 (Supplementary Figure S1).

## Supplementary Materials

The following supporting information can be downloaded at: https://www.mdpi.com/article/10.3390/v14122768/s1. Supplementary Table S1: Assortativity coefficient Data. Supplementary Figure S1: HIV-1 transmission clusters's size distribution identified in RENAGENO dataset.
