# Supplementary material for: Characterization of HIV-1 Transmission Clusters Inferred from the Brazilian Nationwide Genotyping Service Database
Source: Viruses. 2022 Dec 12;14(12):2768. doi: 10.3390/v14122768 (PMC9783618; doi:10.3390/v14122768)
Supplement: Supplementary file 1 [file viruses-14-02768-s001.zip › Supplementary_Figure S1.pdf]

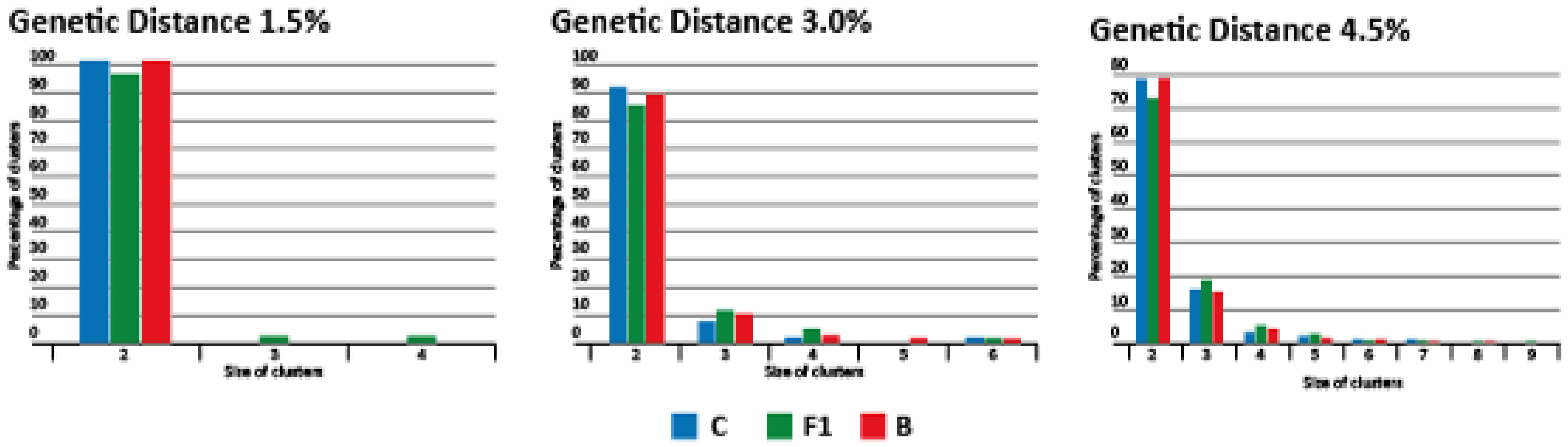

Supplementary Figure S1: HIV-1 transmission clusters's size distribution identified in RENAGENO dataset.
