# Supplementary material for: Characterization of HIV-1 Transmission Clusters Inferred from the Brazilian Nationwide Genotyping Service Database
Source: Viruses. 2022 Dec 12;14(12):2768. doi: 10.3390/v14122768 (PMC9783618; doi:10.3390/v14122768)
Supplement: Supplementary file 1 [file viruses-14-02768-s001.zip › Supplementary_Table S1.pdf]

Supplementary Table S1: Assortativity coefficient Data

Feature	Assortativity	2,50%	97,50%	HIV-1 Subtype	GD (%)
Age	0,763358	-0,3040476	0,2900784	C	1,5
Sampling year	0,6464343	-0,2950129	0,3033291	C	1,5
State	0,7163121	-0,1662727	0,1489362	C	1,5
Municipality	0,5811518	-0,04712042	0,05759162	C	1,5
Years of Education	0,1901408	-0,1755222	0,1678137	C	1,5
Color/Race	0,03030303	-0,1934871	0,1874884	C	1,5
Sex	0,4987469	-0,3032581	0,2982456	C	1,5
Age	0,4761087	-0,1182974	0,115839	C	3
Sampling year	0,3870748	-0,1276233	0,1218036	C	3
State	0,8271231	-0,06429819	0,06171985	C	3
Municipality	0,518914	-0,02247995	0,01798154	C	3
Years of Education		-0,06343124	0,0645739	C	3
Color/Race		-0,07157388	0,07034937	C	3
Sex	0,2815636	-0,1229697	0,1148564	C	3
Age	0,3367494	-0,06706071	0,06376947	C	4,5
Sampling year	0,2790795	-0,06447812	0,07257901	C	4,5
State	0,7009697	-0,03203351	0,02890926	C	4,5
Municipality	0,3848926	-0,011153687	0,009098342	C	4,5
Years of Education		-0,03726404	0,03516681	C	4,5
Color/Race		-0,04188568	0,04214989	C	4,5
Sex	0,2158604	-0,06379225	0,07146053	C	4,5
Age	0,202889	-0,03882671	0,04145048	C	6
Sampling year	0,1680553	-0,03968473	0,0417455	C	6
State	0,6041701	-0,02060803	0,01955929	C	6
Municipality	0,269513	-0,00592544	0,004451957	C	6
Years of Education	0,08413161	-0,02300523	0,02091353	C	6
Color/Race	0,0871304	-0,02409601	0,02391567	C	6
Sex	0,1082911	-0,0384818	0,0410475	C	6
Age	0,1340259	-0,02564637	0,02714926	C	7,5
Sampling year	0,1002708	-0,02484694	0,02475356	C	7,5
State	0,4771032	-0,0126111	0,0126569	C	7,5
Municipality	0,1805339	-0,003933443	0,002499684	C	7,5
Years of Education	0,0582241	-0,01490926	0,0128353	C	7,5
Color/Race	0,05792096	-0,01631207	0,01547263	C	7,5
Sex	0,06767098	-0,02507803	0,02625885	C	7,5
Age	0,7763411	-0,2683539	0,2363506	F	1,5
Sampling year	0,4358461	-0,2476062	0,2450638	F	1,5
State	0,8752688	-0,1047718	0,1006414	F	1,5
Municipality	0,5456682	-0,05438432	0,04297133	F	1,5
Years of Education	0,1787611	-0,1353071	0,1123045	F	1,5
Color/Race	0,08906799	-0,1485326	0,1485033	F	1,5
Sex	0,6888227	-0,2550739	0,2579957	F	1,5
Age	0,6432952	-0,1233566	0,12256	F	3
Sampling year	0,3784867	-0,1195049	0,1132326	F	3
State	0,8029775	-0,04566018	0,04320566	F	3
Municipality	0,5246537	-0,0216536	0,01718197	F	3
Years of Education	0,08123801	-0,06270415	0,05787265	F	3
Color/Race	0,09912233	-0,07723249	0,07511537	F	3
Sex	0,4336215	-0,1160252	0,1128546	F	3
Age	0,4303918	-0,06813419	0,06896506	F	4,5

Sampling year	0,1781181	-0,06494699	0,06446818	F	4,5
State	0,7586088	-0,02532713	0,02378626	F	4,5
Municipality	0,4782514	-0,011001197	0,007582946	F	4,5
Years of Education	0,07712823	-0,03505392	0,03524078	F	4,5
Color/Race	0,1023944	-0,04088841	0,04108737	F	4,5
Sex	0,2946708	-0,06235676	0,05996465	F	4,5
Age	0,3223293	-0,03884313	0,04216971	F	6
Sampling year	0,1773478	-0,03755431	0,04196631	F	6
State	0,6946099	-0,01520041	0,01395439	F	6
Municipality	0,3434091	-0,006690988	0,004820421	F	6
Years of Education	0,04258906	-0,02253728	0,02013836	F	6
Color/Race	0,09576279	-0,02683046	0,02390937	F	6
Sex	0,1781273	-0,04612263	0,03993542	F	6
Age	0,2382978	-0,02532472	0,02462697	F	7,5
Sampling year	0,1313297	-0,02317905	0,02556409	F	7,5
State	0,577734	-0,009703993	0,008143281	F	7,5
Municipality	0,2226608	-0,004327265	0,002609878	F	7,5
Years of Education	0,01384105	-0,01293308	0,012302	F	7,5
Color/Race	0,08486545	-0,01400569	0,01469822	F	7,5
Sex	0,1659425	-0,02659235	0,02410457	F	7,5
Age	0,6653721	-0.2529786	0.2430452	B	1,5
Sampling year	0,5521347	-0.2593525	0.2458690	B	1,5
State	0,8814033	-0.14330787	0.15891602	B	1,5
Municipality	0,6485962	-0,0368952	0,0329854	B	1,5
Years of Education	0,1179941	-0.1078553	0.1137157	B	1,5
Color/Race	0,2949397	-0.084313	0.127467	B	1,5
Sex	0,6192699	-0.1751323	0.1793466	B	1,5
Age	0,5140881	-0.10725747	0.09018227	B	3
Sampling year	0,2625168	-0.09746810	0.10520655	B	3
State	0,792357	-0.06178649	0.05334499	B	3
Municipality	0,5049632	-0,01581028	0,0148563	B	3
Years of Education	0,0703137	-0.06256813	0.06383812	B	3
Color/Race	0,1694234	-0.04089698	0.04386571	B	3
Sex	0,4188582	-0.05887999	0.06326551	B	3
Age	0,3102831	-0.06302067	0.06330889	B	4,5
Sampling year	0,1678345	-0.05564904	0.05294855	B	4,5
State	0,7163489	-0.02942348	0.02886509	B	4,5
Municipality	0,3789621	-0,01552525	0,01515474	B	4,5
Years of Education	0,05282295	-0.04254646	0.04373069	B	4,5
Color/Race	0,1260711	-0.01820196	0.01970844	B	4,5
Sex	0,2645568	-0.04059354	0.03355076	B	4,5
Age	0,1258716	-0,03514288	0,03444514	B	6
Sampling year	0,0829448	-0,02897122	0,02541255	B	6
State	0,6136971	-0,00891265	0,00792155	B	6
Municipality	0,2569841	-0,00498215	0,00405441	B	6
Years of Education	0,0336587	-0,02156145	0,02015488	B	6
Color/Race	0,0842415	-0,01345447	0,01348222	B	6
Sex	0,2154812	-0,03512154	0,03461165	B	6
Age	0,0298451	-0,01267777	0,01365475	B	7,5
Sampling year	0,0459821	-0,01872235	0,01751684	B	7,5
State	0,4884215	-0,00536544	0,00621894	B	7,5
Municipality	0,1722654	-0,002857467	0,002140217	B	7,5
Years of Education	0,0112694	-0,009452154	0,00942145	B	7,5

Color/Race	0,0614781	-0,009872212	0,00972165	B	7,5
Sex	0,1398711	-0,01698225	0,01703258	B	7,5
